# Correlation between vascular endothelial growth factor levels and prognosis of hepatocellular carcinoma patients receiving radiofrequency ablation

**DOI:** 10.1080/13102818.2014.981776

**Published:** 2014-12-01

**Authors:** Qinglong Guan, Junpeng Gu, Haixiao Zhang, Weixin Ren, Weizheng Ji, Yuxiang Fan

**Affiliations:** ^a^ Department of Interventional Radiology, First Affiliated Hospital of Xinjiang Medical University, Wulumuqi, 830054, P. R. China; ^b^ Xinjiang Chinese Medicine Hospital, Huanghe road, Wulumuqi, 830054, P. R. China

**Keywords:** hepatocellular carcinoma, vascular endothelial growth factor, radiofrequency ablation, prognosis

## Abstract

This study is aimed to investigate the effect of serum vascular endothelial growth factor (VEGF) levels on prognosis of hepatocellular carcinoma (HCC) patients receiving radiofrequency ablation (RFA). The 110 HCC patients who received computed tomography (CT) guided RFA were enrolled in this study. The levels of serum VEGF were determined before and after RFA by enzyme-linked immunosorbent assay (ELISA). According to the ELISA results, the patients were divided into the negative group and the positive group. The patient's progression-free survival time was determined. It was demonstrated that the serum VEGF had no significant correlation with ages, sex and tumour size. There were no significant peripheral blood supplies around tumour necrosis. The results showed that higher levels of serum VEGF had a worse prognosis when compared to the patients with lower levels of serum VEGF. The difference between the area under the receiver operating characteristic curve and those when area under curves equalled 0.5 was statistically significant (*P* < 0.05). The serum VEGF level in liver cancer patients can be used as a prognostic indicator for evaluating the efficacy of RFA treatments.

## Introduction

Hepatocellular carcinoma (HCC) is one of the most common malignant tumours worldwide, ranking in the top three in both incidence and mortality. Although there are many treatment options for HCC, most HCC patients are in advanced stage when diagnosed, and lose the chance of surgery. Therefore, the survival rate and prognosis for these HCC patients are usually poor.

In recent years, with the development of imaging equipment and radiofrequency ablation (RFA) instruments, RFA has emerged as a potential alternative to surgical resection for HCC patients. RFA therapy is minimally invasive, more effective, easier to operate, with less complication and able to be repeated, compared with the traditional surgery. RFA not only kills tumour cells but also improves the immunity and reduces the serum concentration of the vascular endothelial growth factor (VEGF). VEGF plays an important role in the vascular formation. VEGF binding to its receptor can facilitate the healing of vascular endothelial, angiogenesis and to increase the vascular wall permeability. Thus, VEGF may be associated with tumour recurrence, metastasis and prognosis.[1]

Studies have shown that growth of liver tumour is dependent on angiogenesis. The proliferation of vascular endothelial cells is induced when the tumour size is greater than 0.5 mm. VEGF is highly expressed in the tumour and is related to the tumour progression and metastasis.[2,3] However, it has not been reported whether the changes in serum VEGF could be used as a prognosis indicator for evaluating the efficacy of the RFA therapy. In this study, 110 HCC patients who received the computed tomography (CT) guided RFA were enrolled. The changes in serum VEGF were determined before and after the RFA therapy, respectively.

## Materials and methods

### General information of patients

A total of 110 HCC patients were selected, of whom 70 cases were males and the other 40 cases were females. The age of patients ranged from 26 to 78 years, and the median age was 60 years. While based on the Barcelona Clinic Liver Cancer (BCLC) staging classification, the stages of HCC patients involved were as following: stage 0, 86 cases; stage A, 19 cases; and stage B, 5 cases. There were 30 patients whose alpha-fetoprotein (AFP) was ≥400 μg/L, and 80 patients whose AFP content was ≤400 μg/L.

Prior written and informed consent was obtained from every patient and the study was approved by the ethics review board of Xinjiang Medical University. Radiofrequency ablation Phillip Big Bore spiral CT was used and the ablation medical equipment was Cool-tip multiple RFA instrument with a punch power of 290 kHz. The different electron needles were selected according to the tumour size and location, and unipolar and multipolar needles were generally used. The lesions were confirmed via imaging before the RFA therapy. The patients took supine or prone position and then the puncture point and needle direction were chosen through CT positioning scanning. When the ablation needle was inserted into the centre of tumour, RFA therapy was carried out by regulating radiofrequency equipment. The ablation time was decided based on the tumour size and location (usually 12 min/site). The position of the electrode needle was adjusted based on the tumour size and shape during the treatment. The ablation range exceeded the original lesion 1–2 cm to make sure the complete ablation of tumour and tumour infiltration.

### Enzyme-linked immunosorbent assay (ELISA)

The peripheral venous blood was collected at one week, one month and three months after the RFA therapy. ELISA was performed to determine the VEGF levels in serum. ELISA kit (Millipore, Billerica, Massachusetts, USA) and the automatic microplate reader were used. The absorbance at 450 nm was measured. The median values of VEGF levels of pre-operative patients and control patients were used as standard values. The patients with VEGF level higher than the median values were defined as the positive group, while the negative groups contained the patients with VEGF level less than the median values.

### Evaluation of efficacies

According to the evaluation criteria set by Union Internationale Contre Le Cancer, treatment efficacy was defined as following: complete remission (CR) meant that lesions completely disappeared; partial remission (PR) meant the mass was reduced more than 50%; stable disease (SD) meant that the tumour size narrowed less than 50% or the tumour size increased less than 25%; progressive disease (PD) meant the size of one or more lesions increased more than 25%.

### Statistical methods

The SPSS17.0 statistical software was used for data analysis. Data were expressed as mean ± standard deviation. Log-rank test was performed for data comparison. The Pearson correlation coefficient was used for analysing the correlation between the prognosis and the factors including pugh score, AFP content, BCLC staging and age, etc. Progression-free survival curves and receiver operating characteristic (ROC) curves were obtained by using Excel to analyse the area under curves (AUC), the sensitivity and the specificity. A *p*-value of <0.05 was considered statistically significant.

## Results and discussion

### General information

As shown in Table 1, 110 HCC patients were enrolled in this study. Among them, 70 were males and 40 were females, with ages ranged between 27 and 68 years. The immunoassay was performed on these patients. The results showed that 17 cases were HBsAg negative (15.4%) and 93 cases were positive (84.5%). In terms of BCLC clinical staging, 86 cases (78.1%) were at stage 0, 19 cases (17.2%) were at stage A and five cases (4.5%) were at stage B. Therefore, the Zps score of the patients was less than grade 1.

**Table 1.** Clinical data of HCC patients before RFA treatment.

Features	Clinical data	Cases (%)
Age (yrs)	<60	32 (29.0%)
	≥60	78 (70.9%)
		
Sex	Male	70 (63.6%)
	Female	40 (36.3%)
		
Child-pugh	A	95 (86.3%)
	B	15 (13.6%)
		
AFP*(μg/L)	<400	80 (72.7%)
	≥400	30 (27.3%)
Tumour size	<2 cm	97 (88.1%)
	2 cm ≤ tumour < 4 cm	13 (11.8%)
Number of tumours	Single	87 (79.1%)
	Multiple	23 (20.9%)
HBsAg	Positive	93 (84.5%)
	Negative	17 (15.4%)
BCLC stage**	0	86 (78.1%)
	A	19 (17.2%)
	B	5 (4.5%)

*Alpha-Fetoprotein. **Barcelona Clinic Liver Cancer.

### The changes in serum VEGF levels of patients before or after RFA

The changes in serum VEGF levels of HCC patients before and after RFA treatment were given in Table 2. The control group included 50 health individuals. The pre-operative VEGF value was 352.1 ± 85.5 pg/mL. This value was decreased to 191.4 ± 24.3 pg/mL within one week after the treatment. The difference was statistically significant (*P* < 0.01) before and after RFA. The VEGF level was increased to 329.9 ± 85.7 pg/mL in one month after RFA. The difference was statistically significant (*P* < 0.01). Furthermore, the VEGF values in three months after the RFA treatment were increased to 493.8 ± 118.4 pg/mL, which was significantly increased compared to the pre-operative serum VEGF (*P* < 0.01). However, the serum VEGF level in the control group was 153.2 ± 30.5 pg/mL only. These results suggest that the VEGF level is elevated after RFA.

**Table 2.** Changes in VEGF levels in serum of HCC patients after RFA.

Time	VEGF (pg/mL)	*r*	*P*
Pre-operative	352.1 ± 85.5		
One week after RFA	191.4 ± 24.3	0.78	<0.01
One month after RFA	329.9 ± 85.7	0.82	<0.01
Three months after RFA	493.8 ± 118.4	0.63	<0.01
Control group	153.6 ± 30.6		

### The relationship among the pre-operative serum VEGF levels, the clinical features and imaging performance

The relationship among the RFA pre-operative serum VEGF levels, the clinical features and imaging performance was analysed. As given in Table 3, the pre-operative serum VEGF levels of the HCC patients receiving RFA had a positive correlation with AFP levels and HBsAg levels. The difference was significant (*P* < 0.05). However, it was demonstrated that the serum VEGF has no significant correlation with age, sex and tumour size (*P* > 0.05).

**Table 3.** Relationships between serum VEGF levels and clinical features.

Clinical features		VEGF (pg/mL)	*r*	*P*
AFP (μg/L)	≥400	442.4 ± 196.0	0.96	0.003
	<400	318.2 ± 169.9		
Tumour size	2 cm ≤ tumour < 4 cm	597.3 ± 133.6	0.14	0.629
	<2cm	314.4 ± 165.4		
Age (yrs)	<60	412.7 ± 98.9	0.28	0.117
	≥60	327.2 ± 105.8		
HBsAg	+	354.0 ± 198.2	0.77	0.001
	–	346.8 ± 45.9		
Sex	Male	390.6 ± 186.7	0.10	0.501
	Female	284.6 ± 163.3		

### Evaluation of efficacy

**Figure 1.**
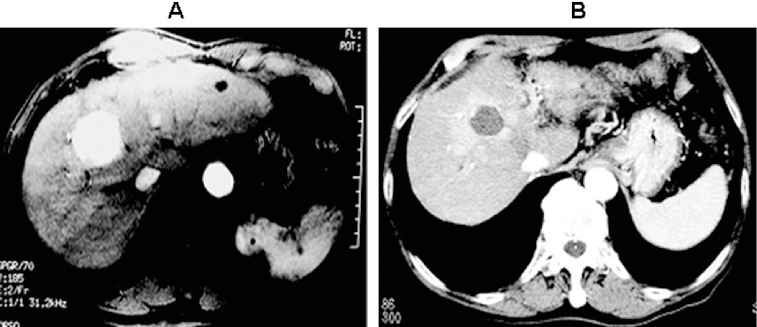
Imaging examination of tumours before and after surgery. (A) Before surgery; (B) after surgery.

According to the tumour size and the tumour edge enhancement assessed by pre-operative and post-operative imaging studies, it was found that there were 84 cases of CR, 17 cases of PR, six cases of SD and three cases of PD. Figure 1 shows that there were obvious differences in tumours before and after RFA treatment. There were no obvious significant peripheral blood supplies around tumour necrosis.

### The effect of serum VEGF levels on the progression-free survival time

**Figure 2.**
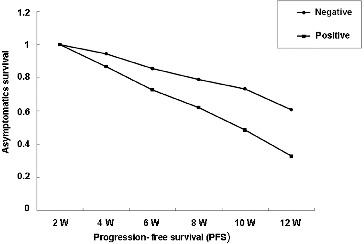
The PFS-asymptomatic survival curves for the positive and negative patients.

The patients were divided into positive group and negative group. In the positive group, the serum VEGF levels of patients were higher than the median values of VEGF, while the serum VEGF of patients in the negative group was less than the median value (Figure 2). At 12 weeks after RFA treatment, the progression-free survival (PFS) time of patients was analysed to understand asymptomatic survival. The results showed that higher levels of serum VEGF had a worse prognosis when compared with the patients with lower levels of serum VEGF (*P* < 0.05, Figure 2).

### The changes in serum VEGF levels of HCC patients before and after RFA

**Figure 3.**
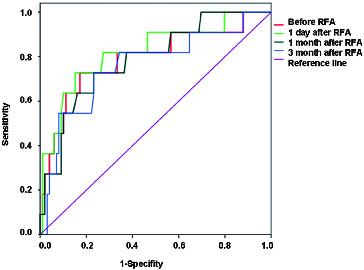
Changes of serum VEGF levels in ROC curve of patients before or after surgery.

According to the measured values of serum VEGF, the ROC curve was generated by using SPSS 17.0 software (Figure 3). The data of sensitivity, specificity and other relevant data were obtained. Based on their complementary relationship, the AUC at one week before RFA, one week after RFA, one month after RFA and three months after RFA were 79.2%, 82.1%, 78.8% and 75.9%, respectively. The difference between the area under the ROC curve and those when AUC equalled to 0.5 was statistically significant (*P* < 0.05). These results suggest that the serum VEGF level in liver cancer patients can be used as a prognostic indicator for evaluating the efficacy of RFA treatments.

### Comparative analysis

RFA treatment for liver cancer causes coagulation necrosis of tumour and improves long-term survival of patients. In this study, 110 patients who received RFA treatment were investigated. The results showed that the PFS rate achieved 90% in six months. Currently, the prognosis after RFA is often analysed through imaging and AFP elimination. However, previous studies have shown that these approaches had poor detection rate in the tumours with residual tumour size less than 0.3 cm after treatment.[4] The possible reason is that AFP has a long half-life in serum, which makes it difficult for the early evaluation of treatment efficacy with AFP. And, there is often no hematological detection index for AFP-negative patients.

The serum VEGF levels began to decline one week after the treatment and the difference was significant (*P* < 0.01) compared to the pre-operative serum VEGF levels. Moreover, the serum VEGF levels were increased in one and three months after treatment. The difference was also significant (*P* < 0.01) by comparing to the pre-operative serum VEGF levels. The changes in serum VEGF levels in one week, one month and three months after RFA may be caused by the reason that ablation leads to the tissue damage and resulting in hypoxia in the surrounding tissues. It, thus, demonstrates that RFA treatment not only kills tumour tissues effectively but also causes cancer cell death. Previous studies show that the transcriptional activity of serum VEGF is related to a hypoxia-inducible factor (HIF-1α) in serum, which plays an important role in VEGF signal transduction.[5–8] Currently, the serum VEGF levels of liver cancer patients can only be measured by ELISA. By combining with other clinical pathological features and the related post-operative examinations, the changes in serum VEGF levels could be helpful for prognosis.[9, 10] Previous studies also demonstrate that tumour size has a great influence on survival rate;[11] however, this study found that the serum VEGF levels in males were slightly higher than in females, but the difference was not significant.

In conclusion, our findings showed that the PFS time of patients with lower serum VEGF levels was significantly better than the patients with higher levels. The pre-operative serum VEGF was calculated to be 347.7 pg/mL by analysing the ROC curves obtained from this study. The results analysis showed that sensitivity and specificity were 97.2% and 90.6%, respectively, indicating that the serum VEGF level is a sensitive and specific factor for prognosis. Therefore, it plays an important role in clinical studies. Our results suggest that the serum VEGF level in liver cancer patients can be used as a prognostic indicator for evaluating the efficacy of RFA treatments.
